# The Endophytic Bacterium, *Sphingomonas SaMR12*, Improves the Potential for Zinc Phytoremediation by Its Host, *Sedum alfredii*

**DOI:** 10.1371/journal.pone.0106826

**Published:** 2014-09-08

**Authors:** Bao Chen, Jianguo Shen, Xincheng Zhang, Fengshan Pan, Xiaoe Yang, Ying Feng

**Affiliations:** 1 MOE Key laboratory of Environment Remediation and Ecosystem Health, College of Environmental and Resource Sciences, Zhejiang University, Zijingang Campus, Hangzhou, China; 2 Agricultural Ecology and Plant Protection Management Station, Yuhang County, Hangzhou, China; UMass, United States of America

## Abstract

The endophytic bacterium, *Sphingomonas SaMR12*, isolated from *Sedum alfredii* Hance, appears to increase plant biomass and zinc-extraction from contaminated soil; however, the mechanism by which this occurs is not clear. Here, the ability of *SaMR12* to promote zinc extraction and its effects on root morphology and exudation were examined in hydroponics. Zinc treatment increased shoot biomass by 30 to 45%, and by a further 10 to 19% when combined with *SaMR12* inoculation. Zinc treatment also increased zinc accumulation modestly and this too was enhanced with *SaMR12*. Both biomass and zinc levels increased in a dose-dependent manner with significant effects seen at 50 µM zinc and apparent saturation at 500 µM. Zinc and the endophyte also increased levels of auxin but not at 50 µM and zinc increased levels of superoxide and hydrogen peroxide but mainly at 500 µM. As for root morphology, *SaMR12* increased root branching, the number of root tips, and surface area. Zinc and *SaMR12* also increased the exudation of oxalic acid. For most assays the effects of the endophyte and zinc were additive, with the notable exception of *SaMR12* strongly reducing the production of reactive oxygen species at 500 µM zinc. Taken together, these results suggest that the promotion of growth and zinc uptake by exposure to zinc and to *SaMR12* are independent of reactive oxygen and do not involve increases in auxin.

## Introduction

Zinc is an essential trace element that can have toxic effects on organisms at millimolar levels through soil or water contamination. Excessive zinc is toxic to plants and negatively affects human health [1], [2]. Phytoremediation represents a thorough, economical, and environmentally friendly method compared to conventional technology, that can absorb heavy metals from soil and transport them from the root to shoot, decreasing the soil concentration of metals [3]. According to Barceló et al. [4], two important aspect of phytoremediation are phyto-extraction, which denotes reducing the concentration of heavy metal in soil by a hyperaccumulator taking up the metal, and phyto-stabilization, which denotes stabilizing pollutants in soil by a plant that converts them to less available forms.

For successful phytoremediation, to overcome the shortcomings of low biomass and low growth rate, various approaches have been examined, such as identifying hyperaccumulators, fertilizing soil to improve plant biomass, and applying organic acid or chelators to increase metal bioavailability [5]–[8]. Plant-derived chelators, known as phytochelatins, are produced by roots (and include organic and amino acids as well as their derivatives) and often in quantities that are linearly correlated with the ambient level of heavy metal.

A member of the Crassulaceae, *Sedum alfredii* is a plant found in an ancient, Chinese mining region near Quzhou (Zhejiang province) and has a strong ability to extract heavy metals from soil. In previous studies [5], [9], [10], *S. alfredii* was found to show heavy metal resistance and accumulation, as well as the ability to efficiently transport heavy metals from the root to the shoot. Roots of *S. alfredii* mainly produce malic acid, oxalic acid, and tartaric acid, presumably contributing to enhanced bioavailability of heavy metals [11].

However, root exudates not only improve access to metals they also significantly affect microbial growth and community structure within the rhizoshpere [12]. Microbes in the rhizosphere facilitate plant growth, enhance nitrogen absorption through the biological fixation of nitrogen, and promote root elongation and the formation of root hairs [13]. Taghavi et al. [14] found that endophytic bacteria can promote plant root development and significantly improve shoot growth. Meharg and Killham [15] found that soil microorganisms can increase or decrease root exudation modestly but, arguably, enough to affect rhizosphere microbial processes.

According to a previous study, *S. alfredii* roots are host to at least four species of endophytic bacteria, and when this plant is inoculated with one of them, *Sphingomonas SaMR12*, plant biomass and heavy metal absorption are both significantly increased [16]. However, the mechanisms of this enhancement are not clear. Particularly unclear are the effects of *SaMR12* on heavy metal bioavailability and root exudation. According to Přikryl and Vančura [17], inoculation of *Pseudomonas putida* enhanced the release of root exudates. Therefore, here, we hypothesize that, in *Sedum alfredii, SaMR12* affects root morphology and organic acid composition of root exudates, thereby altering zinc bioavailability and rhizosphere conditions, and leading to larger plant biomass and higher zinc extraction.

## Materials and Methods

### Experiment design and materials

*Sedum alfredii* Hance, a zinc/cadmium hyperaccumulator identified previously [9], was obtained from an old lead and zinc mining area in Zhejiang Province, China. The work described here did not involve endangered or protected species. Specific permissions were not required for collection of samples in this location.

The endophytic bacterium *Sphingomonas SaMR12*, previously isolated from *S. alfredii* [16], was grown on solid Luria-Bertani (LB) medium at 37°C overnight. A single bacterial clone was cultured in LB liquid medium overnight. The cells was collected by centrifugation and washed three times with sterile water.

Healthy shoots of *S. alfredii* were collected in the field and cultured in Hoagland nutrient solution for 2 months in 2.5 L (10.2-cm top diameter) standard round green plastic pots, aerated continuously and renewed every week. Single shoot tips were excised and grown as described above for another month to remove heavy metals. Plants showing uniform growth were treated with the indicated concentrations of zinc, with 6 plants per pot for hydroponic experiments. Supplemental zinc was supplied as ZnSO_4_·7H_2_O and added to maintain the zinc concentration every three days. Unsupplemented Hoagland's contains 5 µM zinc (considered here as the ‘control’ level of zinc). A second group of plants was subjected to the same treatment and also inoculated with *SaMR12* at densities of 10^4^ to 10^5^ CFU/mL. Each treatment was repeated three times and the experiment lasted for one month.

### Measurement of root morphological parameters

At the end of the one-month experiment, roots were washed with distilled water three times, photographed by a digital camera (D3000, Nikon, Tokyo, Japan), scanned using an automatic root scanner (STD1600, Seiko Epson Corp., Japan), and analyzed using WinRHIZO software (Version 3.9, Regents Instruments Inc., 2001, Quebec, Canada). Additionally, 3 cm apical root segments were imaged through a light microscope (Eclipse E600W, Nikon), from which root hair morphology was assessed.

### Measurement of biomass and zinc concentration

At the end of the experiment, shoots and roots were separated and weighed. The roots were washed in several changes of 5 mM Tris-HCl pH 6.0 containing 5 mM EDTA, and were then washed with distilled water to remove non-specifically bound zinc. Shoots and roots were oven-dried at 80°C to a consistent weight. For subsequent analysis, oven-dried samples were ground using a stainless steel mill (MM400, Retsch, Haan, Germany) to an average particle size of 0.5 mm. Next, the ground material was digested using acid HNO_3_-HClO_4_ (v/v, 5∶1) for 8 h at 180°C before subjecting the samples to inductively coupled plasma-mass spectroscopy (7500a, Agilent, Santa Clara, CA, USA). To ensure accuracy of determination, a standard zinc solution was run first to calibrate the instrument.

### Root exudates collection and examination

Root exudates were collected according to the method of Ma et al. [18]. Briefly, the root systems of intact plants were placed in 100 mL of 0.5 mM CaCl_2_ for 4 h. The solution was passed through a resin column [Amberlite IR–120 (H^+^ form)] and then through another resin column [Dowex 1 × 8 100–200 (Cl^−^ form)]. Exudates were dried using a rotary evaporator and resolved in methanol before high-pressure liquid chromatography (HPLC) analysis (Agilent 1200 series, Waldbronn, Germany). Organic acids were detected at 210 nm by UV detection by comparing retention times and absorption spectra with organic acid standards.

### Assay for indole-3-acetic acid (IAA) and active oxygen generation

IAA concentration in the plant was determined according to the method described by Hou et al. [19] with modifications. First, 5 g of young leaves were ground to a fine powder with liquid nitrogen and IAA was extracted with 20 mL 80% methanol at 4°C overnight. The liquid phase was obtained by centrifugation at 10,000×*g* for 10 min and extracted two additional times with a 10 mL extraction solution for 1 h. After concentration, liquid partition, column separation, and elution, the eluent was passed through a sterile 0.2 µm filter prior to injection into the HPLC.

The generation of intracellular reactive oxygen species (ROS) was analyzed by measuring the superoxide anion as described by Nag et al. [20] and H_2_O_2_ as described by Tang et al. [21], using an ultraviolet spectrophotometer (Lambda 35, PerkinElmer, Waltham, MA, USA).

### Statistical analyses

Data were compared by least significant difference (LSD) tests at a 5% significance level. Two-way analysis of variance was performed to determine the effects of *SaMR1*2 inoculation (*S*), zinc concentration (Zn), and inoculation × zinc concentration (*S* × Zn) on root morphology using the SPSS software (version 20.0, SPSS Inc., Chicago, IL, USA). All figures were produced using Origin (version 8.5, OriginLab, Northampton, MA, USA).

## Results

### Effect of *SaMR12* on *S. alfredii* growth and zinc uptake

**Figure 1:**
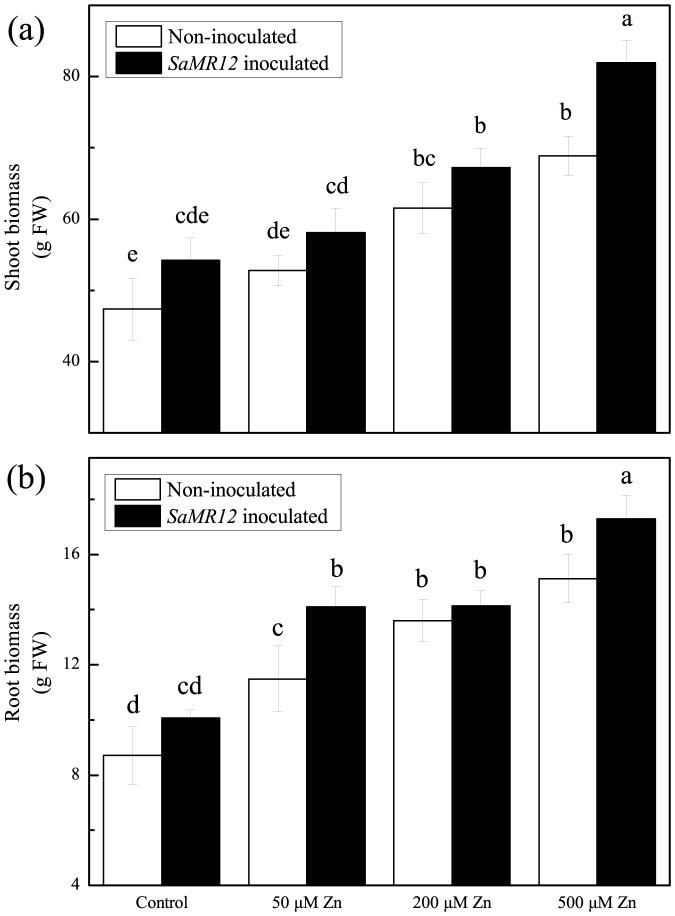
Shoot and root biomass of *S. alfredii* affected by zinc and *SaMR12* treatment. Bars plot mean ± SD of three replicate experiments. The different letters above the bars indicate significant differences among treatments at the *P*<0.05 level.

Both zinc concentration and *SaMR12* inoculation significantly stimulated plant growth (Fig. 1). Shoot biomass significantly increased with zinc concentration, increasing by 30% on 200 µM zinc and by 45% on 500 µM zinc. At all Zinc concentrations, inoculation with *SaMR12* also promoted shoot growth significantly, and the effect appeared to be additive with that of zinc. Similar results were observed for root biomass (Fig. 1b).

**Figure 2:**
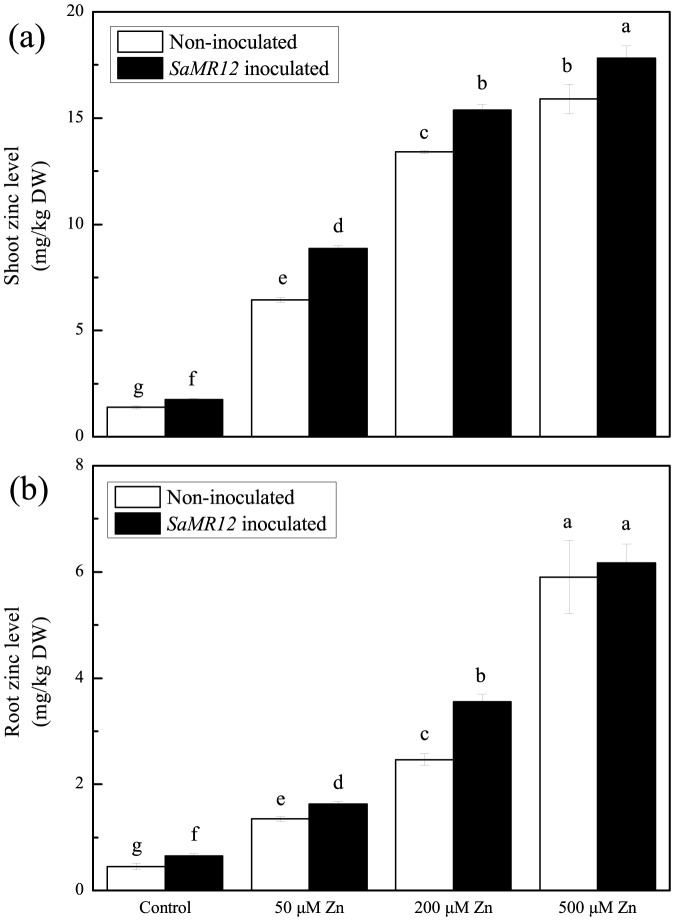
Zinc concentration in the plant affected by *SaMR12* and the zinc treatment. Bars plot mean ± SD of three replicate experiments. Letters show significance as for Figure 1.

**Figure 3:**
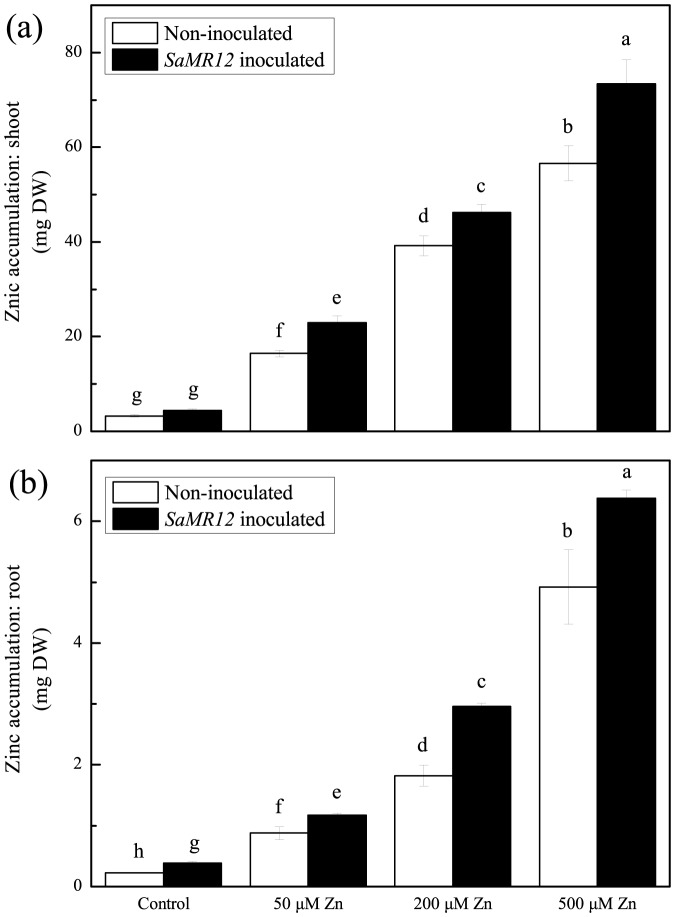
Zinc accumulation affected by *SaMR12* and zinc treatment. Bars plot mean ± SD of three replicate experiments. Letters show significance as for Figure 1.

Zinc supply also significantly affected the concentration and accumulation of zinc within the plant (Figs. 2 and 3). Over the range of tested levels, the zinc concentration increased ∼11-fold for shoots (Fig. 2a) and ∼13-fold for roots (Fig. 2b). For treatments that included *SaMR12*, zinc concentration increased modestly but significantly at all treatment levels for the shoot (Fig. 2a) and at all levels except for the highest for the root (Fig. 2b). Reflecting the increased biomass, these patterns were closely reflected by absolute zinc amount in both shoots and roots (Fig. 3). In fact, on 500 µM zinc, total zinc amount was increased 17 times at least that of the control in both organ system, and the presence of the bacterium the increase was closer to 23 fold.

### Plant IAA concentration as affected by *SaMR12* and zinc treatment levels

**Figure 4:**
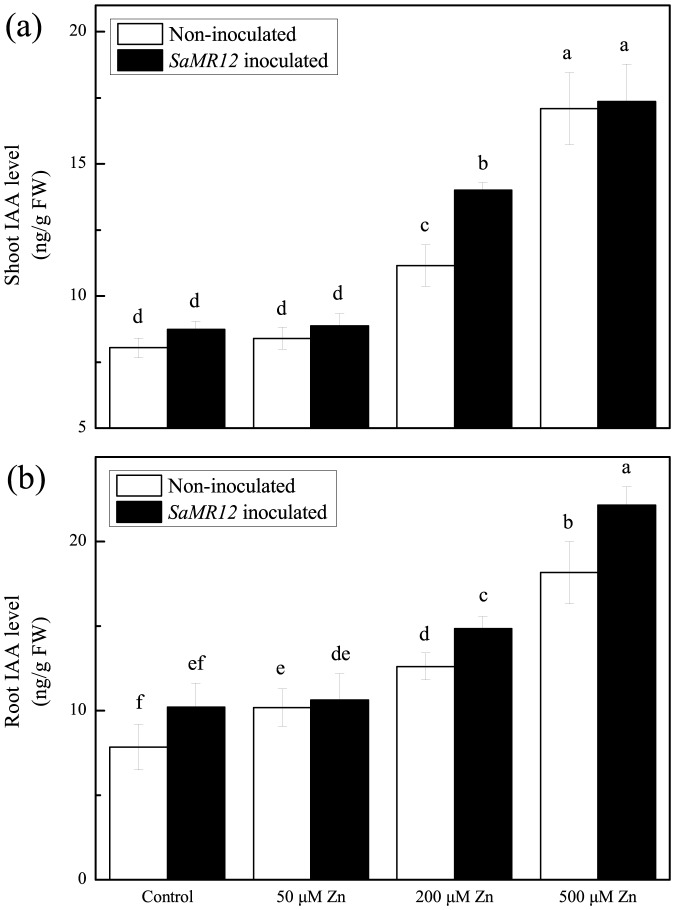
IAA concentration as affected by *SaMR12* and zinc treatment. Bars plot mean ± SD of three replicate experiments. Letters show significance as for Figure 1.

Zinc did not alter the concentration of IAA in the shoot up to 50 µM, but shoot IAA concentration significantly increased by 33% on 200 µM zinc, and by 104% on 500 µM (Fig. 4a). These levels were increased further by *SaMR12* inoculation only at 200 µM, where the increase was 26% more than the non-inoculated. In contrast, roots appeared slightly more sensitive, increasing IAA level at all tested zinc concentrations, and increasing even more with inoculation, except at 50 µM (Fig. 4b). The relative increase in IAA was similar in the two organ systems although the magnitude of the increases in the shoot seemed to be somewhat larger.

### Effect of zinc and *SaMR12* treatment on oxidative stress

**Figure 5:**
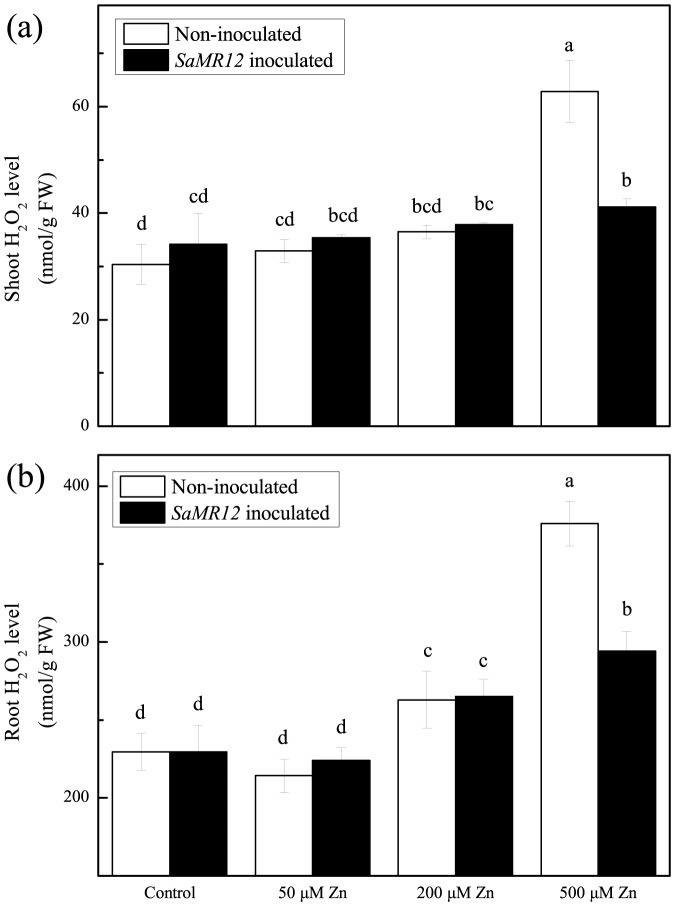
H_2_O_2_ concentration as affected by *SaMR12* and the zinc treatment. Bars plot mean ± SD of three replicate experiments. Letters show significance as for Figure 1.

**Figure 6:**
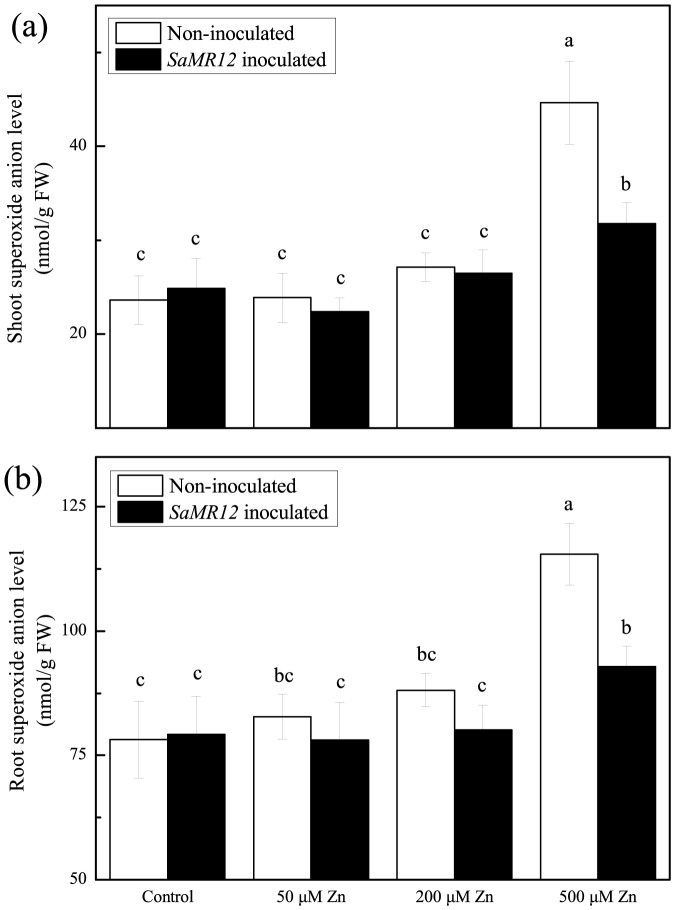
Superoxide anion concentration as affected by *SaMR12* and the zinc treatment. Bars plot mean ± SD of three replicate experiments. Letters show significance as for Figure 1.

Zinc is an important component of the reactive oxygen scavenging system as a part of copper/zinc-superoxide dismutase; in addition, the element can also aggravate oxidative stress through increased production of reactive oxygen. Here, to examine the role of reactive oxygen in this system, we quantified the levels of hydrogen peroxide and superoxide in shoots and root (Figs. 5 and 6). Up to and including 200 µM, neither species changed appreciably (not at all in the shoot and only slightly in the root) and there was no alteration in level caused by the bacterium. However, at 500 µM zinc, both anions more or less doubled in level in both organs and in all cases inoculation with the bacterium reduced these increases to almost control levels. These results suggest that reactive oxygen species have little or nothing to do with the enhanced growth and metal accumulation seen here.

### Effect of zinc and *SaMR12* on root system morphology

**Figure 7:**
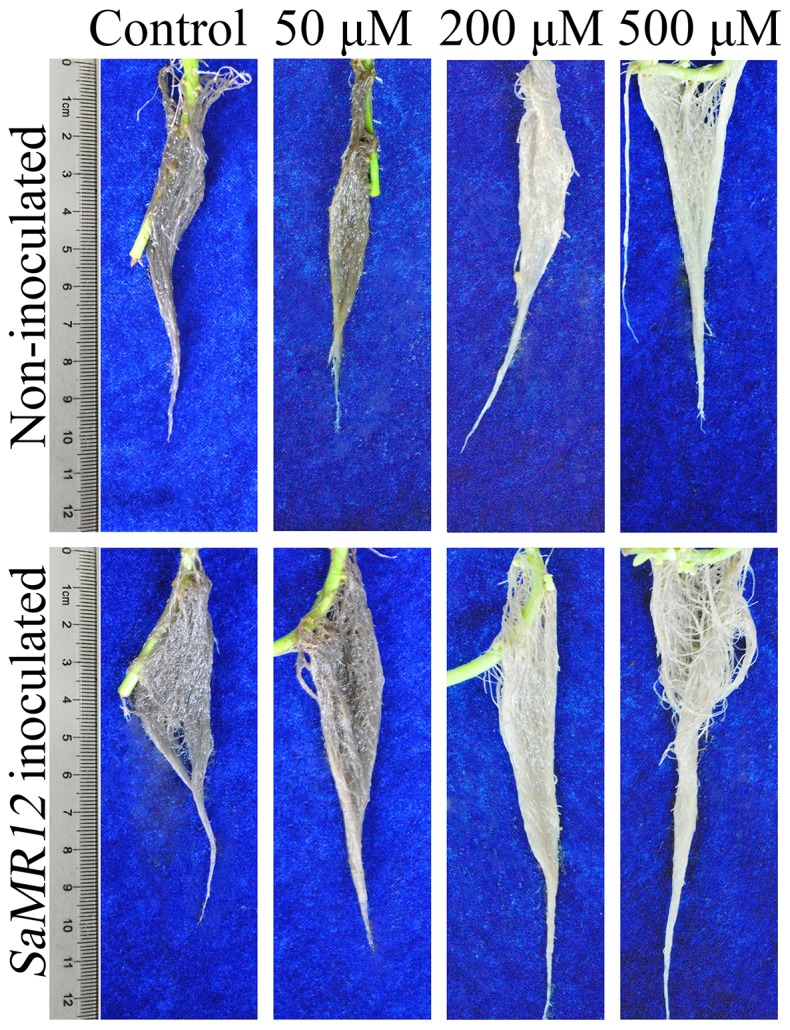
Root architecture as affected by *SaMR12* and the zinc treatment. Representative images.

Both zinc and *SaMR12* treatments enhanced root growth (Fig. 7). When the zinc concentration was 200 or 500 µM, the roots appeared more healthly, with longer roots, larger biomass, and lighter color. The total root length, number of root tips, and root surface area were significantly promoted with increasing zinc treatment concentration (Table 1). After inoculation with *SaMR12*, the root biomass was further increased compared to non-inoculated treatment at the same zinc levels (Fig. 1), and total root length was increased by 12 to 26%, root tip number by 24 to 33%, and surface area by 17 to 32% (Table 1).

**Table 1:** Root parameters and main and interactive effects of zinc and *SaMR12* on root tips, root total length, and root surface area of *S. alfredii*.

Treatments		Root tips (tips plant^−1^)		Total root length (cm plant^−1^)		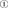 Surface area (cm^2^ plant^−1^)	
Control	Non-inoculated	647±53 f		785±88 d		166±9 e	
	*SaMR12* inoculated	809±31 e		986±30 c		203±16 d	
50 µM	Non-inoculated	647±48 f		785±36 d		191±11 d	
	*SaMR12* inoculated	857±51 e		964±90 c		224±11 c	
200 µM	Non-inoculated	940±32 d		1069±63 c		208±7 cd	
	*SaMR12* inoculated	1210±24 b		1202±70 b		257±10 b	
500 µM	Non-inoculated	1113±13 c		1233±54 b		222±3 c	
	*SaMR12* inoculated	1377±44 a		1440±54 a		292±6 a	
Two-way analysis of variance							
Source of variation		*F*	*P*	*F*	*P*	*F*	*P*
	*SaMR12* (*S*)	199.1	0.000**	47.6	0.000**	140.3	0.000**
	Zn level (Zn)	247.5	0.000**	71.9	0.000**	61.7	0.000**
	Interaction (*S* × Zn)	2.465	0.100	0.418	0.743	4.158	0.023*

Values represent the mean ± standard deviation of three replicates.

^*^Significant at the *P*<0.05 level,

^**^Significant at the *P*<0.01 level.

The different letters following the values in the same column indicate significant differences between the treatments at *P*<0.05 (Duncan's test). Main and interactive effects of zinc and *SaMR12* on the root tips, root total length and root surface area of *S. alfredii* were examined by two-way analysis of variance. Both *SaMR12* and zinc treatment significantly affected root tips (*F* = 199.2, *P*<0.01), total length (*F* = 47.6, *P*<0.01), and total surface area (*F* = 140.3, *P*<0.01) of *S. alfredii* (*P*<0.01). The total surface area of the root was also significantly affected by the interactive effects of (*S* × Zn) at the *P*<0.05 level.

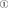
The surface areas did not include root hairs which cannot be detected using the root automatic scan apparatus.

**Figure 8:**
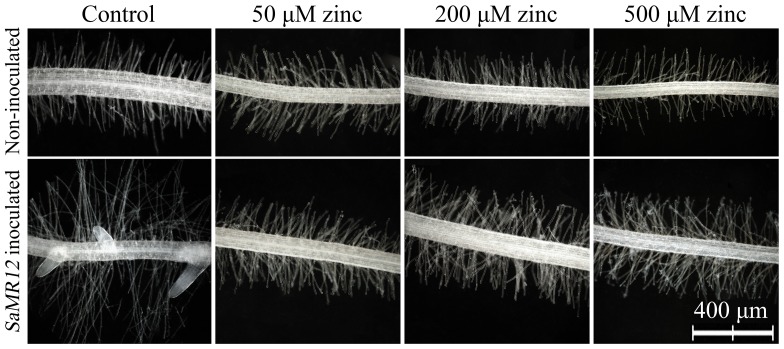
Root hairs affected by *SaMR12* and zinc treatment. Representative images.

Regarding root hairs, zinc treatment alone caused little conspicuous alteration except that on 500 µM zinc they appeared to be less numerous (Fig. 8). After inoculation with *SaMR12*, root hair length and lateral root formation both appeared to be strongly increased under control conditions (i.e., 5 µM zinc) but at the higher concentrations of zinc, lateral root formation was not stimulated and root hairs were more numerous and only slightly longer than that of the respective non-inoculated treatment control.

### Effect of zinc and *SaMR12* on root exudates

**Figure 9:**
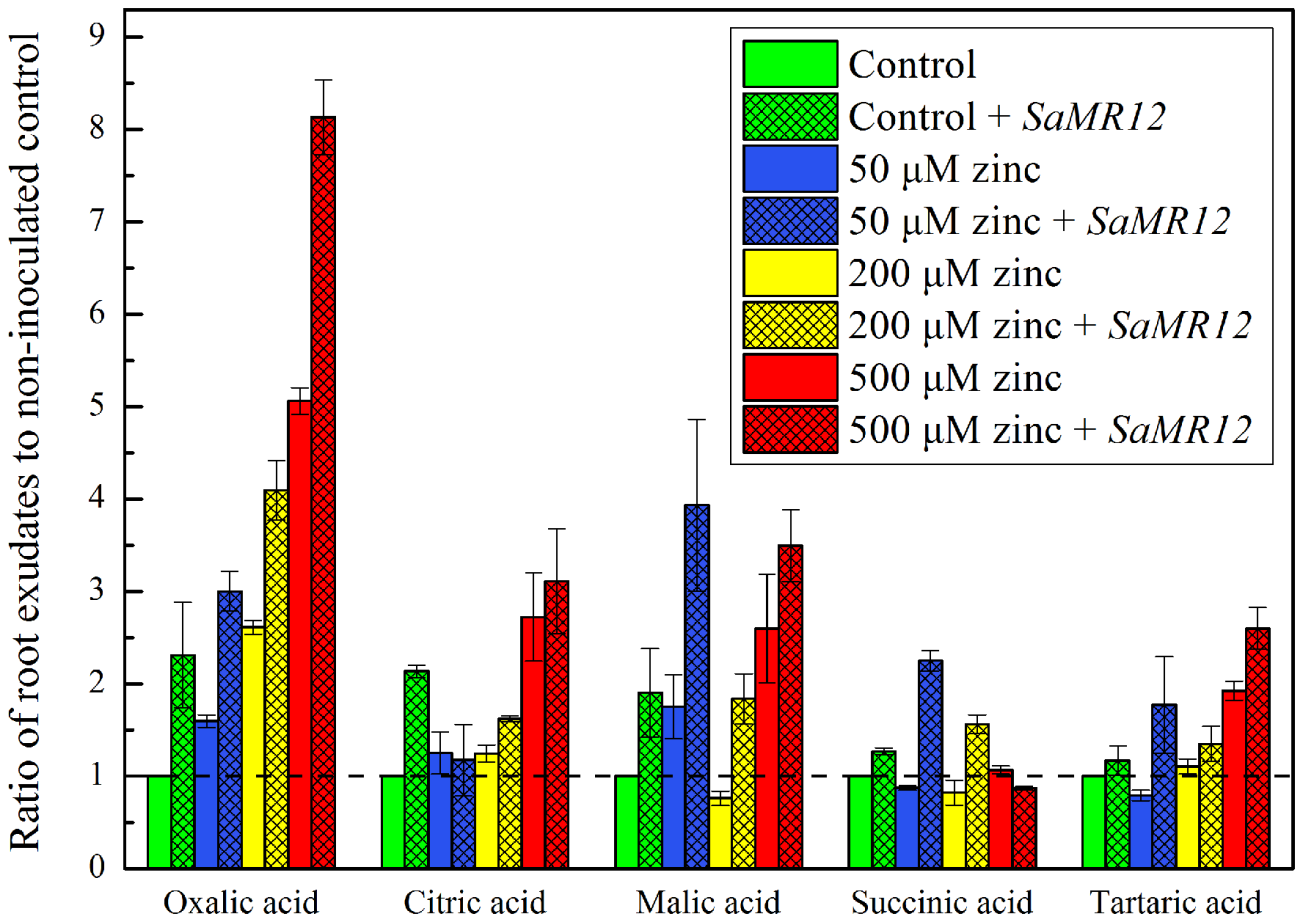
Organic acid exudation as affected by *SaMR12* and zinc treatment. Bars plot mean ± SD of three replicate experiments. The level of each acid is normalized to the value obtained for the non-inoculated and un-supplemented treatment. Raw data are shown in table 2.

Organic acids in root exudates were detected by HPLC. Absolute values and significance are shown in Table 2 but for ease of understanding, the data are shown graphically in Fig. 9, normalized against the values for non-inoculated control. Succinate exudation was scarcely affected at any treatment, as were tartarate and citrate, except at 500 µM zinc when they were roughly doubled, regardless of the presence of inoculum. The pattern of malate exudation was more complex, being stimulated to about the same extent (2 to 4 fold) on 50 and 500 µM zinc but being little affected on 200 µM. In contrast, oxalate exudation showed a clear response to zinc, increasing in a dose-dependent way and being substantially larger with inoculation. The pattern of oxalic acid exudation resembles that of biomass increase as well as zinc accumulation and suggests that the exudation of this acid plays a pivotal role in zinc acquisition.

**Table 2:** Root exudates affected by zinc and *SaMR12* inoculation.

Treatment		Organic acid (mg/kg h, FW)				
		Oxalic acid	Citric acid	Malic acid	Succinic acid	Tartaric acid
Control	Non-inoculated	15.9±1.06 f	15.1±0.96 bc	53.2±8.72 d	61.2±5.76 b	31.2±3.45 bc
	*SaMR12* inoculated	32.3±6.99 cd	27.7±0.68 a	88.9±4.78 bc	66.2±1.56 b	30.5±1.13 bc
50 µM	Non-inoculated	19.5±2.6 ef	14.6±2.79 bc	67.6±6.79 cd	40.3±2.36 c	22.4±1.02 d
	*SaMR12* inoculated	29.3±1.62 de	18.5±0.64 b	123.1±5.77 a	84.1±4.28 a	28.6±2.46 bcd
200 µM	Non-inoculated	26.5±1.31 de	12.0±1.01 c	57.9±5.15 d	32.2±4.42 c	23±0.76 d
	*SaMR12* inoculated	39.9±2.48 bc	15.9±0.87 bc	61.1±7.49 d	58.2±1.92 b	25.9±1.89 cd
500 µM	Non-inoculated	46.3±3.02 b	23.6±2.89 a	75.9±3.32 bcd	37.4±2.95 c	35.3±2.29 b
	*SaMR12* inoculated	64.9±3.71 a	12.3±0.98 c	91.6±11.52 b	35.4±4.55 c	45.4±2.3 a

Values represent the mean ± standard deviation of three replicates. ^*^Significant at the *P*<0.05 level, ^**^Significant at the *P*<0.01 level.

The different letters following the values in the same column indicate significant differences between the treatments at *P*<0.05 (Duncan's test).

## Discussion

During phytoremediation, it is an important for plants to continually absorb heavy metals from the soil and transport them from the root to the shoot. Three factors are necessary for this process to be effective: (1) the plants must be resistant to heavy metal; (2) the plants must absorb metals from soil; (3) the metal should be transported from the root to the shoot [22]. In aiding phytoremediation, endophytic bacteria can produce IAA, 1-aminocyclopropane-1-carboxylate deaminase, siderophore, or promote phosphate solubilization and nitrogen fixation [23], [24]. These are important mechanisms for alleviating metal stress, promoting nutrient absorption, and providing growth-promoting substances [25]–[28]. For instance, Chen et al. [29] found that plant growth-promoting endophytes significantly increased *Solanum nigrum* biomass and enhanced cadmium extraction from the soil. In a previous study, the endophytic bacterium *SaMR12* was shown to be effective for promoting growth of the host plant [16]. Here, we found that *SaMR12* affects plant growth, root morphology, oxidative stress, and root exudates.

The biomass of a plant is a primary factor limiting successful remediation of heavy metal contamination. In sugar beet, above-ground, endophytic bacteria increased photosynthetic efficiency by increasing chlorophyll content, leading to increased carbohydrate synthesis [30]. In red pepper, Islam et al. [31] found a significant increase in chlorophyll content after inoculation with a nitrogen-fixing bacterium. And in micropropagated sugarcane, Oliveira et al. [32] proved that inoculation endophytic diazotrophic species resulted in 30% increasing of total plant nitrogen content. Underground, a large area of contact between the root and soil is necessary to increase absorption of the metals from the soil; as such, remediation efficiency can be increased through an enlarged root system [33], [34].

Here, following inoculation with the endophytic bacterium, root length, number, and surface area increased significantly (Table 1), which agrees with the results of a previous study [35]. There has been speculation that these changes are driven by auxin, produced either directly by the endophyte or indirectly by the plant in response. For example, the roots of canola were more than 35% longer following treatment with wild-type *P. putida* GR12-2 compared with non-inoculated control, while the IAA-deficient mutant did not show this increase [36]. Various studies have demonstrated that IAA-producing bacteria promoted plant growth to some extent [37]. The black truffle fungus, *Tuber melanopsorum*, induces alterations in both host (*Cistus incanus*) and non-host (*Arabidopsis thaliana*) roots, such as promoting root hair elongation, that are reminiscent of altered auxin levels [38], [39]. Nevertheless, although we find that zinc and the endophyte *SaMR12* increase auxin levels, the concentration dependence differed from the promotion of biomass and zinc accumulation. Both of the latter were clearly stimulated on 50 µM zinc whereas there was little increase in IAA. Furthermore, root hairs responded vigorously to the presence of endophyte but this response seemed more or less independent of the level of zinc (Fig. 8). Taken together, these results imply that IAA increases are secondary or even inconsequential for the action of zinc and endophyte in promoting biomass and metal accumulation in *S. alfredii*.

Zinc is typically not considered to induce reactive oxygen in plants, however, various studies have demonstrated that zinc can activate copper/zinc superoxide dismutase and proline production to alleviate metal-induced oxidative stress or enhance the activities of antioxidant enzymes [40], [41]. In this study, the concentrations of H_2_O_2_ and superoxide were little changed until the zinc treatment concentration reached 500 µM, and at that concentration, the oxidative stress of the inoculated plants was found to be ameliorated compared with non-inoculated plants (Figs. 5 and 6). Similar effects of endophytes on ryegrass and *Achnatherum inebrians* (drunken horse grass) have been reported [42], [43]. However in *S. alfredii*, it remains unclear whether the observed alleviation is a consequence of better nutrition or a direct action of the endophyte, such as the production of anti-oxidative enzymes or siderophore.

Root exudates comprise a complex set of compounds, which help mobilize mineral nutrients and condition the rhizosphere [44]. According to a review by Hinsinger et al. [45], root exudates improve metal element availability by decreasing rhizosphere pH, potentially by making absorption by the plant easier [46]. However, distinct organic acids have distinct efficacy. Wu et al. [47] found that the sequence of organic acids that solublize heavy metals is: malate ≥ citrate ≈ oxalate for zinc; and malate ≥ citrate > oxalate for cadmium. For various plants, Cieśliński et al. [48] found that the high cadmium accumulator secreted significantly more low-molecular-weight organic acids than the low cadmium accumulator in three soil types, and the extractable soil cadmium and cadmium accumulation in plants gradually increased with higher low-molecular-weight organic acid production.

In a previous study, the exogenous addition of citric or tartaric acid significantly increased the availability and absorption of cadmium [46]. Here, the root secreted oxalic acid as a function of zinc concentration, and this secretion rate was further increased when the plant was inoculated with *SaMR12*. Higher zinc (500 µM) treatment stimulated citric acid secretion, which agreed with the results of Saber et al. [49], who found for sunflower a marked increase in the excretion of malate and citrate with exposure to zinc or aluminum, and high concentrations of malic and citric acids alleviated the toxicity effect of these metals on plant growth. In *Pinus sylvestris*, oxalic acid was stimulated by exposure to elevated aluminum and was found to be significantly higher in mycorrhizal-treated plants than in non-mycorrhizal controls [50].

The activating effect of organic acid on heavy metal uptake is commonly related to pH. Succinate enhanced the mobilization of arsenic, copper, lead, and zinc under neutral pH, but inhibited this mobilization under acidic conditions [51]. Cadmium-resistant tomato plants were not susceptible to cadmium stress because of oxalate secretion by the root apex, which can form a precipitate with cadmium to efficiently ameliorate toxicity [52]. In contrast, Wu et al. [53] found that oxalate, citrate, or malate in the soil at the same concentrations had virtually no effect on metal uptake by Indian mustard (*Brassica juncea*).

## Conclusion

This is apparently the first study to investigate the effects of inoculation with the endophytic bacterium, *SaMR12*, on plant growth, root morphology, and root exudates, a study that might facilitate the understanding of mechanisms of microbe-assisted phytoremediation. Through hydroponic experiments, *SaMR12* inoculation significantly enhanced the efficiency of zinc phyto-extraction by increasing *S. alfredii* biomass, promoting zinc absorption, improving root morphology, and enhancing root exudates. Further we could show that although IAA levels and reactive oxygen species rose, both could be plausibly disconnected from the promoting action of both zinc itself and the endophyte. The application of endophytes to phytoremediation not only strengthens the ability of phyto-accumulators to acclimate to the contaminated environment, but also expands the application range of phytoremediation.
